# Supplementary material for: Hierarchical Molecular Graph Self-Supervised Learning for property prediction
Source: Commun Chem. 2023 Feb 17;6:34. doi: 10.1038/s42004-023-00825-5 (PMC9938270; doi:10.1038/s42004-023-00825-5)
Supplement: Supplementary file 2 — Supplementary Information [file 42004_2023_825_MOESM2_ESM.pdf]

Supplementary Information

Hierarchical Molecular Graph Self-supervised Learning for Property Prediction

Xuan Zang[1], Xianbing Zhao[1] and Buzhou Tang[1,2*]

[1]Department of Computer Science, Harbin Institute of Technology, Shenzhen, 518055,China.
[2] Pengcheng Laboratory, Shenzhen, 518055, China.

*Corresponding author(s). E-mail(s): tangbuzhou@gmail.com

Supplementary Note 1 - Graph neural networks

Given a graph $G = (V, E)$, GNN aims to learn the representation of each node $v \in V$ according to the adjacency information derived by E. GNN implements the message passage mechanism, which iteratively updates the node representation by aggregating neighbor features. The message passage mechanism consists of two main steps: AGGREGATE and COMBINE. AGGREGATE intends to aggregate the information from neighbors of nodes; COMBINE is to update node features by integrating the aggregated neighbor information. $L-$layer GNN can aggregate $L-$hop neighbors. The lth GNN layer is formalized as:

$$\mathbf{h}_v^l = \text{COMBINE}^l \left(\mathbf{h}_v^{l-1}, \text{AGGREGATE}^l \left(\left\{ h_u^{l-1} : u \in \mathcal{N}(v) \right\} \right) \right) \tag{1}$$

where $\mathcal{N}(v)$ is the neighbor node set of v, $\mathbf{h}_v^l$ is the hidden feature of node v after l-layer aggregation, and $\mathbf{h}_v^0$ is the input feature of node v. Additionally, the READOUT operation is conducted to get the entire graph representation, which pools the aggregated node representation.

$$\mathbf{h}_G = \text{READOUT} \left(\left\{ \mathbf{h}_v^L \mid v \in V \right\} \right) \tag{2}$$

For instance, sum, max, mean and attention functions are general READOUT operations.

Supplementary Note 2 - Self-supervised learning

Self-supervised learning (SSL) conducts pre-training on a large-scale unlabeled dataset, then the pre-trained model is transferred to downstream tasks to perform fine-tuning with a small number of groundtruth. Benefiting from the advantages of processing large-scale unlabeled data, SSL has been widely used in computer vision (CV) and Natural Language Processing (NLP). Besides, the past few years have witnessed the success of SSL in graph learning. Graph SSL can be classified into three main categories according to self-supervised signals: contrast, generative, and predictive[1, 2].

- Contrast graph SSL constructs different views by graph data augment and takes the commonality and difference information between inter-data pairs as supervision signals. Namely, aligning the representations of positive view pairs and differentiating the representations of negative view pairs. The construction of views is the key point of contrast graph SSL.
- Generative graph SSL generally takes the graph structure itself as the supervision signal, with the purpose of reconstructing topology structure or masked attributes. Graph auto-encoder and graph auto-regression are two fundamental approaches to generating graph structure.
- Predictive graph SSL creates some pseudo-labels with simple statistical analysis or expert knowledge. Then prediction tasks are designed based on these

generated pseudo-labels. Self-supervised molecular graph learning constructs labels in the light of unique chemical structures, such as motifs.

Supplementary Note 3 - Detailed description of baselines

- GraphSAGE[3] introduces an inductive method to aggregate neighbor information, the trained mapping of nodes to embedding can handle unseen nodes. During pre-training, neighbor pairs and non-neighbor pairs are sampled as positive and negative samples, and SSL is conducted in light of edge prediction.
- GPT_GNN[4] employs a probabilistic generative model to generate graph structure and attributes. Specifically, some edges and node features are masked, attribute generation predicts masked node attributes through observed data; edge generation reconstructs unobserved edges.
- AttributeMask[5] randomly masks some edge attributes and leverages GNN to generate the masked attributes.
- ContextPred[5] constructs the context subgraph, which is encoded as a vector by context GNN. Meanwhile, the main GNN aggregates the neighborhood information to obtain the embedding of the center node. The objective of context prediction is to make the embedding obtained by aggregation neighbors more similar to that of the same node encoded on the context graph.
- InfoGraph[6] contrasts graph-level representation obtained by READOUT function and all patch-level representations, in which a node and the graph it belongs to is a positive sample pair, and negative samples are formed by it and the other graphs in the identical batch.
- MoCL[7] combines two different contrast strategies. The local-level strategy contrasts representations encoded by the two graph augmentations; the global-level strategy contrasts mutual information between similar graph pairs.
- GraphLoG[8] preserves local similarity through the alignment of similar subgraphs and introduces hierarchical prototypes to achieve global semantic structure.
- GraphCL[9] obtains two L-hop subgraphs with random perturbations for a node and conducts self-supervised learning by maximizing the similarity between the two subgraphs.
- JOAO[10] design a framework to automatically select data augmentation methods on the basis of GraphCL. The general idea is to train iteratively the probability matrix of multiple data augmentation methods through adversarial training, and correspondingly replace the projection head in GraphCL.
- MolCLR[11] three methods of molecular augmentations, including atom masking, bond deletion, and subgraph removal, and contrasts different augmentation methods.
- G_Motif[12] proposes a dynamic messaging passing network based on Transformer and considers motif prediction task as the self-supervised signal.

- MGSSL[13] improves the rules of motif construction, uses GNN backbone to encode molecular graph representation, and predicts the motifs based on a given order (depth-first search or breadth-first search) on the graph.

Supplementary Method

For multi-level pretext tasks, learnable Multi-layer Perceptrons (MLP) ϕ_{link}, ϕ_{atom_type}, ϕ_{bond_type}, ϕ_{atom_num}, and ϕ_{bond_num} are used to decode the predicted values $\hat{y}_{ij}$, $\hat{y}_{v,k}$, $\hat{y}_{e,k}$, $\hat{y}_a$, and $\hat{y}_b$. Formally, we give the the input, output and the detailed layers of all MLPs as follows.

$$
\begin{aligned}
&concat[\mathbf{h}_i, \mathbf{h}_j] \rightarrow \phi_{link}\{Linear(2d, d) \rightarrow Relu \rightarrow Linear(d, 1)\} \rightarrow \hat{y}_{ij} \\
&\mathbf{h}_v \rightarrow \phi_{atom\_type}\,\{Linear(d, d) \rightarrow Relu \rightarrow Linear(d, N_{atom\_type})\} \rightarrow \hat{y}_{v,k} \\
&concat[\mathbf{h}_i, \mathbf{h}_j] \rightarrow \phi_{bond\_type}\,\{Linear(2d, d) \rightarrow Relu \rightarrow Linear(d, N_{bond\_type})\} \rightarrow \hat{y}_{e,k} \\
&\mathbf{H}_{\mathrm{g}} \rightarrow \phi_{atom\_num}\,\{Linear(d, d/4) \rightarrow Softplus \rightarrow Linear(d/4, 1)\} \rightarrow \hat{y}_a \\
&\mathbf{H}_{\mathrm{g}} \rightarrow \phi_{bond\_num}\,\{Linear(d, d/4) \rightarrow Softplus \rightarrow Linear(d/4, 1)\} \rightarrow \hat{y}_b
\end{aligned}
\tag{3}
$$

where *concat* is the concatenation operation, $N_{atom\_type} = 118$ and $N_{bond\_type} = 4$ are the number of atom types and bond types.

Supplementary Table 1 Input feature type and range of nodes and edges. The atomic indices of graph-level and motif-level nodes are 119 and 120 respectively. Their degrees are set to 0. For both types of nodes, node-motif and motif-graph edges are augmented. Their features of Bond is_in_ring are None.

Feature category	Feature type	Range
Node feature	Atom type	[1,118]+[119,120]
	Atomic degree	[0,10]
Edge feature	Bond type	{single,double,triple,aromatic}+{node-motif, motif-graph}
	Bond is_in_ring	{False, True}+{None}

Supplementary Table 2 Detailed summary of all downstream and pre-training datasets used in HiMol.

Datasets	Molecules	Tasks	Task Type	Metrics	Avg_nodes	Avg_degree
BACE	1,522	1	classification	ROC-AUC	34.1	2.2
BBBP	2,053	1	classification	ROC-AUC	24.1	2.1
Tox21	8,014	12	classification	ROC-AUC	18.6	2.0
ToxCast	8,615	617	classification	ROC-AUC	18.8	2.0
SIDER	1,427	27	classification	ROC-AUC	33.6	2.0
ClinTox	1,491	2	classification	ROC-AUC	26.2	2.1
ESOL	1,128	1	regression	RMSE	13.3	2.1
FreeSolv	643	1	regression	RMSE	8.7	1.8
Lipophilicity	4,200	1	regression	RMSE	27.0	2.2
QM7	7,165	1	regression	MAE	6.8	1.9
QM8	21,786	12	regression	MAE	7.8	2.1
QM9	133,885	12	regression	MAE	8.8	2.1
ZINC15	249,456	-	-	-	23.2	2.1

Supplementary Table 3 Comparison of self-supervised pattern between our HiMol and baselines.

Baselines	Generative	Contrast	Predictive
GraphSAGE[3]	✓	-	-
GPT_GNN[4]	✓	-	-
AttributeMask[5]	✓	-	-
ContextPred[5]	-	✓	-
InfoGraph[6]	-	✓	-
MoCL[7]	-	✓	-
GraphLoG[8]	-	✓	-
GraphCL[9]	-	✓	-
JOAO[10]	-	✓	-
MolCLR[11]	-	✓	-
G_Motif[12]	-	-	✓
MGSSL[13]	-	-	✓
HiMol	✓	-	✓

Supplementary Table 4 Summary of hyperparameter on all downstream datasets during fine-tuning of HiMol. lr_feat and lr_pred denote the learning rate of GNN backbone and MLP for prediction, respectively.

Datasets	batch_size	epoch	lr_feat	lr_pred	dropout	dimension
BACE	32	100	1e-3	1e-3	0.5	512
BBBP	32	100	5e-4	1e-3	0.5	512
Tox21	32	100	5e-4	1e-3	0.7	512
ToxCast	32	100	1e-3	1e-3	0.7	512
SIDER	32	100	5e-4	1e-3	0.5	512
ClinTox	32	100	1e-3	1e-3	0.5	512
ESOL	32	100	1e-3	1e-3	0.5	512
FreeSolv	32	100	1e-3	1e-3	0.5	512
Lipophilicity	32	100	5e-4	5e-4	0.5	512
QM7	32	100	1e-3	1e-3	0.5	512
QM8	32	100	1e-3	1e-3	0.5	512
QM9	32	100	1e-3	1e-3	0.5	512

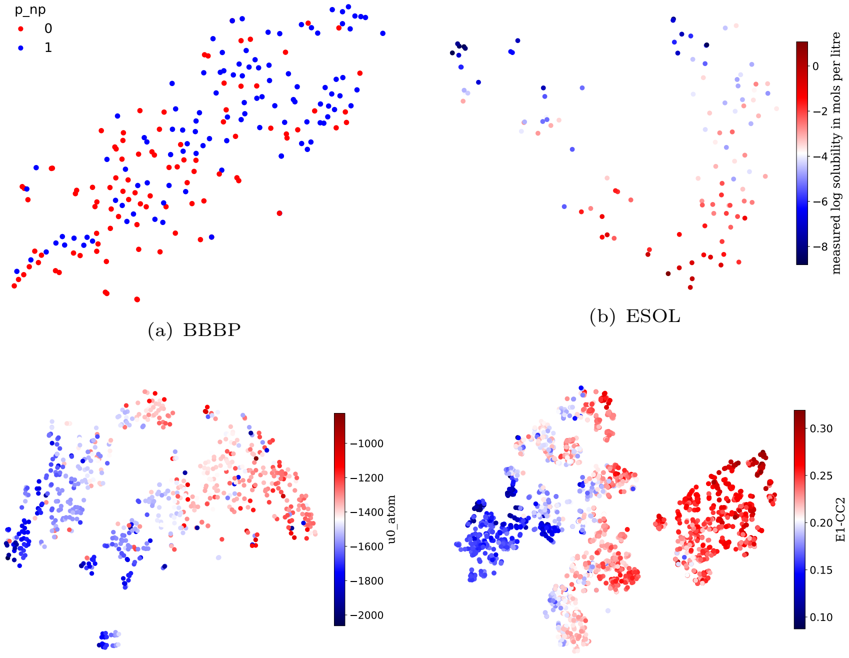

(a) BBBP

(b) ESOL

(c) QM7

(d) QM8

Supplementary Figure 1 Visualization of molecular representations obtained by our HiMol on the downstream test set.(a) For BBBP, blue color represents penetration and red color represents non-penetration. (b) For ESOL, color represents the measured water solubility of compound. (c) For QM7, color represents electronic property of molecule. (d) For QM8, color represents the transition energy modeled by CC2 method.

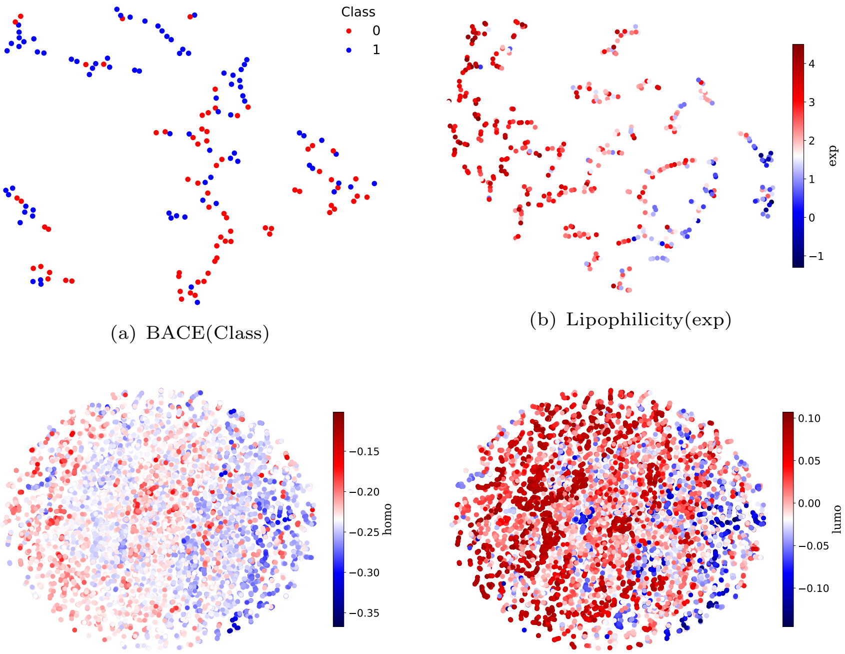

(a) BACE(Class)

(b) Lipophilicity(exp)

(c) QM9(homo)

(d) QM9(lumo)

Supplementary Figure 2 Visualization of molecular representations without pre-training on the downstream test set. (a) For BACE(Class), color represents binary labels of binding results for BACE-1 inhibitors. (b) For Lipophilicity(exp), color represents octanol/water distribution coefficient. (c) For QM9(homo), color represents highest occupied molecular orbital energy (homo) of molecules. (d) For QM9(lumo), color represents lowest unoccupied molecular orbital energy (lumo) of molecules.

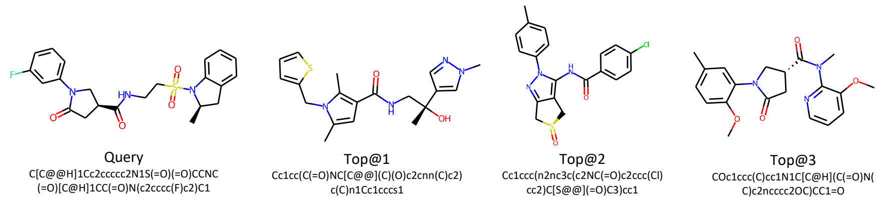

(a) ZINC13968356

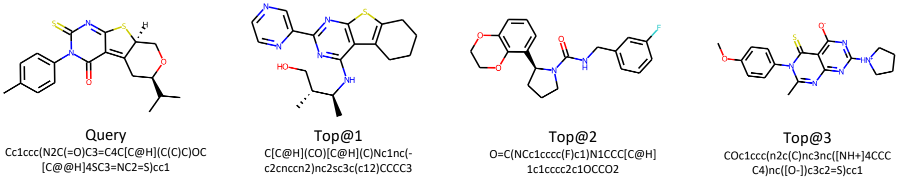

(b) ZINC38926049

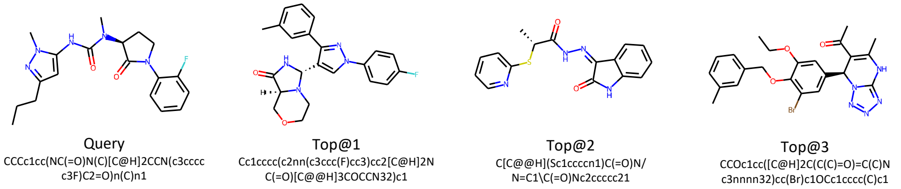

(c) ZINC91091439

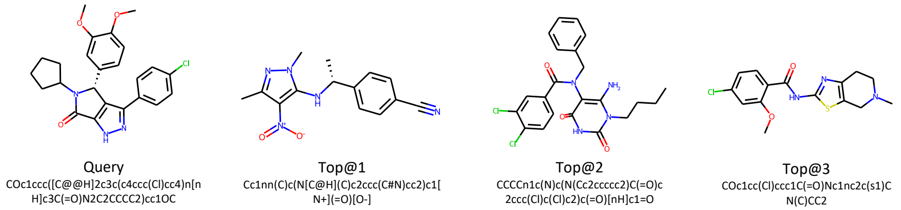

(d) ZINC40342449

Supplementary Figure 3 Visualization of the top-three molecules ranked by molecular representation similarity for the four query molecules. SMILES for all molecules are given.

Supplementary References

[1] Wu, L., Lin, H., Tan, C., Gao, Z., Li, S.Z.: Self-supervised learning on graphs: Contrastive, generative, or predictive. IEEE Transactions on Knowledge and Data Engineering (2021)

[2] Liu, Y., Jin, M., Pan, S., Zhou, C., Zheng, Y., Xia, F., Yu, P.: Graph self-supervised learning: A survey. IEEE Transactions on Knowledge and Data Engineering (2022)

[3] Hamilton, W., Ying, Z., Leskovec, J.: Inductive representation learning on large graphs. Advances in neural information processing systems **30** (2017)

[4] Hu, Z., Dong, Y., Wang, K., Chang, K.-W., Sun, Y.: Gpt-gnn: Generative pre-training of graph neural networks. In: Proceedings of the 26th ACM SIGKDD International Conference on Knowledge Discovery & Data Mining, pp. 1857–1867 (2020)

[5] Hu, W., Liu, B., Gomes, J., Zitnik, M., Liang, P., Pande, V., Leskovec, J.: Strategies for pre-training graph neural networks. In: International Conference on Learning Representations (2019)

[6] Sun, F.-Y., Hoffman, J., Verma, V., Tang, J.: Infograph: Unsupervised and semi-supervised graph-level representation learning via mutual information maximization. In: International Conference on Learning Representations (2019)

[7] Sun, M., Xing, J., Wang, H., Chen, B., Zhou, J.: Mocl: data-driven molecular fingerprint via knowledge-aware contrastive learning from molecular graph. In: Proceedings of the 27th ACM SIGKDD Conference on Knowledge Discovery & Data Mining, pp. 3585–3594 (2021)

[8] Xu, M., Wang, H., Ni, B., Guo, H., Tang, J.: Self-supervised graph-level representation learning with local and global structure. In: International Conference on Machine Learning, pp. 11548–11558 (2021)

[9] You, Y., Chen, T., Sui, Y., Chen, T., Wang, Z., Shen, Y.: Graph contrastive learning with augmentations. Advances in Neural Information Processing Systems **33**, 5812–5823 (2020)

[10] You, Y., Chen, T., Shen, Y., Wang, Z.: Graph contrastive learning automated. In: International Conference on Machine Learning, pp. 12121–12132 (2021)

[11] Wang, Y., Wang, J., Cao, Z., Barati Farimani, A.: Molecular contrastive learning of representations via graph neural networks. Nature Machine

Intelligence **4**(3), 279–287 (2022)

[12] Rong, Y., Bian, Y., Xu, T., Xie, W., Wei, Y., Huang, W., Huang, J.: Self-supervised graph transformer on large-scale molecular data. Advances in Neural Information Processing Systems **33**, 12559–12571 (2020)

[13] Zhang, Z., Liu, Q., Wang, H., Lu, C., Lee, C.-K.: Motif-based graph self-supervised learning for molecular property prediction. Advances in Neural Information Processing Systems **34**, 15870–15882 (2021)
